# The European response to control and manage multi- and extensively drug-resistant *Neisseria gonorrhoeae*

**DOI:** 10.2807/1560-7917.ES.2022.27.18.2100611

**Published:** 2022-05-05

**Authors:** Michelle J Cole, Michaela Day, Susanne Jacobsson, Andrew J Amato-Gauci, Gianfranco Spiteri, Magnus Unemo, , the European Gonorrhoea Response Plan Group, European Gonorrhoea Response Plan Group, Eszter Balla, Birgit van Benthem, Tatjana Nemeth Blažić, Maria José Borrego, Helen Fifer, Lilly Grothier, Steen Hoffmann, Gwenda Hughes, Catherine Ison, Klaus Jansen, Robert D. Kirkcaldy, Otilia Mårdh, Ndeindo Ndeikoundam, Teymur Noori, Raj Patel, Teodora Wi

**Affiliations:** 1 UK Health Security Agency, London, United Kingdom; 2 World Health Organization Collaborating Centre for Gonorrhoea and other STIs, Örebro University, Örebro, Sweden; 3 European Centre for Disease Prevention and Control, Stockholm, Sweden; 4 University College London (UCL), London, United Kingdom; 5 The members of the network are listed under Collaborators

**Keywords:** antimicrobial resistance, cephalosporins, EU/EEA, gonorrhoea, MDR-NG, surveillance, treatment failure, XDR-NG

## Abstract

Because cefixime and ceftriaxone resistance in *Neisseria gonorrhoeae* and gonorrhoea treatment failures were increasing, a response plan to control and manage multidrug-resistant *N. gonorrhoeae* (MDR-NG) in Europe was published in 2012. The three main areas of the plan were to: (i) strengthen surveillance of antimicrobial resistance (AMR), (ii) implement monitoring of treatment failures and (iii) establish a communication strategy to increase awareness and disseminate AMR results. Since 2012, several additional extensively drug-resistant *N. gonorrhoeae* (XDR-NG) strains have emerged, and strains with high-level ceftriaxone resistance spread internationally. This prompted an evaluation and review of the 2012 European Centre for Disease Prevention and Control (ECDC) response plan, revealing an overall improvement in many aspects of monitoring AMR in *N. gonorrhoeae*; however, treatment failure monitoring was a weakness. Accordingly, the plan was updated in 2019 to further support European Union/European Economic Area (EU/EEA) countries in controlling and managing the threat of MDR/XDR-NG in Europe through further strengthening of AMR surveillance and clinical management including treatment failure monitoring. The plan will be assessed biennially to ensure its effectiveness and its value. Along with prevention, diagnostic, treatment and epidemiological surveillance strategies, AMR surveillance is essential for effective control of gonorrhoea.

## Background

Gonorrhoea is the second most commonly reported bacterial sexually transmitted infection (STI) in the European Union/European Economic Area (EU/EEA), with 117,881 cases reported in 2019, representing an increase of 17% since 2018 [1]. In the absence of a vaccine, conventional prevention including condom use, early diagnosis together with antimicrobial treatment are the main public health strategies to interrupt transmission and to avoid sequelae such as pelvic inflammatory disease, ectopic pregnancy and infertility [2]. Effective treatment of gonorrhoea is unfortunately hindered by the fact that *Neisseria gonorrhoeae* (NG) has developed antimicrobial resistance (AMR) to all classes of therapeutic antimicrobials, most recently to third-generation cephalosporins [3]. Surveillance of NG AMR in the EU/EEA is performed by the European Gonococcal Antimicrobial Surveillance Programme (Euro-GASP) [4]. Euro-GASP is funded and coordinated by the European Centre for Disease Prevention and Control (ECDC) and includes a network of mostly reference laboratories in the EU/EEA countries. The main aim of Euro-GASP is to measure annual prevalence of and trends in gonococcal antimicrobial susceptibility to detect emerging AMR and to inform optimisations of national and international treatment guidelines [4]. Additional linked activities include an external quality assessment (EQA) programme, training, performance of EU/EEA-wide molecular epidemiological studies and provision of evidence-based practices, guidance and responses to emerging STI concerns [5]. A review of Euro-GASP representativeness revealed that the Euro-GASP data mainly reflect national measures of the AMR situation for *N. gonorrhoeae* across Europe, but the degree of representativeness is affected by suboptimal isolate numbers and low completeness of reporting [6].

As a response to the emergence of in vitro resistance to cefixime and to treatment failures with cefixime and subsequently ceftriaxone in the EU/EEA and internationally [3], ECDC and the Euro-GASP coordinating hub together with an extended international expert group developed the evidence-based *Response plan to control and manage the threat of multidrug-resistant gonorrhoea in Europe* in 2012 [7]. Similar response plans were published by the World Health Organization (WHO) [8] and by national public health agencies in many countries, e.g. by the United Kingdom (UK) Health Security Agency (UKHSA) (formerly Public Health England and Health Protection Agency) [9] and by the United States Centers for Disease Control and Prevention [10]. In addition, gonorrhoea management guidelines were updated in Europe and internationally to recommend dual antimicrobial therapy regimens of an intramuscular dose of ceftriaxone (250–500 mg) plus an oral dose of azithromycin (1–2 g) [11].

Regular monitoring of the gonococcal AMR situation and AMR response plan indicators, and evaluating, reviewing and subsequent updating of national and international response plans are essential. Since the 2012 ECDC response plan [7] was published, Euro-GASP has detected decreasing levels of cefixime resistance from 8.7% in 2010 to 1.4% in 2018 [1]. In contrast, there were reports of ceftriaxone-resistant isolates again (three isolates in 2018 vs none in 2016 and 2017). Azithromycin resistance (using the recently removed European Committee on Antimicrobial Susceptibility Testing (EUCAST) azithromycin clinical resistance breakpoint of > 0.5 mg/L [12]) also increased from 4.5% in 2013 to 13.3% in 2018, and isolates with high-level resistance to azithromycin (MIC ≥ 256 mg/L) have been detected in several EU/EEA countries [1]. Ciprofloxacin resistance has consistently remained high (over 46%), although this drug was discontinued for the treatment of gonorrhoea many years ago [1]. Furthermore, the first global treatment failure with a dual-therapy regimen, caused by an extensively drug-resistant (XDR)-NG strain, was confirmed in the UK in 2016 [13]. In 2018, the first XDR-NG strain with ceftriaxone resistance and high-level azithromycin resistance, acquired in South-East Asia, was reported in the UK [14]. This strain resulted in treatment failure for pharyngeal gonorrhoea with 1 g intramuscular dose of ceftriaxone. Two isolates of the same XDR-NG strain were subsequently identified in 2018 in Australia, and one of these patients had a travel link to South-East Asia [15]. The simultaneous detection of the same XDR-NG strain from opposite sides of the globe prompted the release of a rapid risk assessment by ECDC [16]. Along with the requirement to enhance antimicrobial susceptibility testing (AST) in many regions and ensure ongoing collaboration between the gonococcal AMR surveillance programmes globally, the detection of these isolates highlighted the importance of appropriately noted travel history for patients with gonorrhoea. Furthermore, since 2015, a ceftriaxone-resistant strain has been spreading internationally and resulted in ceftriaxone treatment failures in several countries, including in the EU/EEA [17].

Decreasing azithromycin susceptibility and sporadic resistance to ceftriaxone threaten the effectiveness of dual-therapy and ceftriaxone monotherapy regimens [18]. Ceftriaxone is the last remaining option for empiric monotherapy of gonorrhoea and, with the occurrence of XDR-NG, the threat of untreatable gonorrhoea has emerged. Therefore, in 2019, the Euro-GASP network and ECDC decided to update the 2012 ECDC response plan [7]. 

Our aim was to describe the evaluation of the 2012 ECDC response plan, by analysing the progress of indicators from 2012 to 2017, as well as the subsequent review and update of the response plan to further support EU/EEA countries in implementing national strategies and interventions to control the threat of multidrug-resistant (MDR)- and XDR-NG in a multidisciplinary approach.

## 2012 ECDC response plan and evaluation

The main goal of the 2012 ECDC response plan was to mitigate the impact of MDR-NG on the prevention and control of gonorrhoea by serving as a guide for EU/EEA countries [7]. Three main areas of focus were detailed in the plan; (i) strengthening surveillance to obtain timely AMR data with sufficient epidemiological data, (ii) implementing treatment failure monitoring and (iii) establishing a communication strategy to increase awareness of MDR-NG and to appropriately disseminate AMR surveillance results.

To evaluate the effectiveness of the 2012 ECDC response plan [7], members of the Euro-GASP network were asked to provide information on previously agreed indicators. The year 2012 was used as the baseline and progress for each indicator was evaluated against the situation in 2017. A questionnaire with the indicators was distributed to the National Focal Points for HIV, STI and Hepatitis and ECDC National Coordinators across the EU/EEA, as well as the Euro-GASP laboratory network (n = 31 countries) with 22 countries providing a response (Table 1). The majority of countries had a national GASP (n = 18) and STI clinic network (n = 19), along with a platform to disseminate the AMR data (n = 16). Laboratory capacity was assessed by 14 respondents: however, only six had national training modules available. Therefore, national and local capacity building for gonococcal AST may be limited and could subsequently impact the quality and reliability of the AST data. Accordingly, training should continue to be a focus of the Euro-GASP network to ensure that appropriate capacity for quality-assured culture and AST in EU/EEA countries is available or further developed. Data regarding clinical management were less encouraging, with 11 or fewer countries stating they had reviewed clinical management guidelines, could report treatment failures and were using their own or ECDC treatment failure case definitions. More than half (n = 13) of the countries had published AMR and/or MDR-NG data, with fewer countries stating that they had a national communication plan (n = 7) and fact sheets (n = 9) available (Table 1). Potential unsuccessful patient management was mitigated by the use of European guidance at the national level as many countries stated that they still applied the 2012 European guideline for gonorrhoea management [19], the 2012 ECDC response plan [7] and the EU/EEA case definitions [20]. Management of gonorrhoea treatment failures, however, was still a gap across the EU/EEA which the updated 2019 ECDC response plan will further focus on and improve.

**Table 1:** Indicator responses from Euro-GASP participating countries, 2017 (n = 22^a^)

Component	Indicator	Number of countries where indicator was met
Strengthen surveillance	National gonococcal antimicrobial surveillance programme in place	18
	STI clinic network established (sentinel or other)	19
	National platform for sharing of information/data on gonorrhoea AMR established	16
	Assessment of laboratory capacity performed	14
	National training modules (laboratory and/or clinical) available	6
Clinical management	Case definitions for gonorrhoea treatment failure agreed and implemented	10
	National gonorrhoea treatment failure reporting/monitoring implemented	9
	Gonorrhoea clinical management guidelines reviewed and revised	11
	Recommended culture and AMR testing for cases of suspected treatment failure	17
Communication strategy	National communication plan agreed	7
	Fact sheet adjusted and disseminated	9
	National publications or communications on MDR *Neisseria gonorrhoeae*	13

AMR: antimicrobial resistance; Euro-GASP: European Gonococcal Antimicrobial Surveillance Programme; MDR: multidrug-resistant; STI: sexually transmitted infection.

^a^ Responses received from Austria, Belgium, Croatia, Cyprus, Denmark, Estonia, Finland, Germany, Hungary, Iceland, Latvia, Liechtenstein, Luxembourg, Malta, the Netherlands, Norway, Portugal, Romania, Slovakia, Slovenia, Sweden and the United Kingdom.

We also assessed indicators related to Euro-GASP (Table 2). The number of countries participating in Euro-GASP AMR surveillance and EQA was higher in 2017 compared with 2012 (27 vs 20 for AMR surveillance and 28 vs 15 for EQA), along with a 41% increase in the number of isolates submitted to Euro-GASP, mainly attributable to large increases in isolate numbers from five countries [1]. Because of the large overall increase in the number of gonorrhoea cases in Europe, the proportion of isolates submitted to Euro-GASP among the overall number of reported gonorrhoea cases at the EU/EEA level was, however, similar (3.7% in 2012 and 3.6% in 2017). In respect to reporting of Euro-GASP epidemiological variables, good completeness (i.e. > 90%) was recorded for the variables age, sex and site of infection in both 2012 and 2017, but there was limited or no improvement in reporting for other variables, including sexual orientation. This is an important area that needs further improvement and which Euro-GASP continuously works on, so that better analysis can take place. Timeliness of reporting Euro-GASP results was suboptimal for the 2012 data as the report was not published until 2014. Therefore, the annual Euro-GASP report has been redeveloped into a more concise report and data are now also available in the ECDC Surveillance Atlas of Infectious Diseases [1] within 6 months of reporting. Only two treatment failures meeting the case definition were officially reported to ECDC between 2012 and 2017; one additional case was investigated, but it did not fit the case definition.

**Table 2:** Euro-GASP indicators, 2012 vs 2017

Component	Indicator	Indicators 2012 [1]	Indicators 2017 [1]	Indicator achieved/progress
Strengthen surveillance	Number of countries participating in Euro-GASP	20/30	27/31	Increased by seven countries
	Number of isolates reported through Euro-GASP	1,927 (3.7% of reported gonorrhoea cases)	3,248 (3.6% of reported gonorrhoea cases)	Increased by 41% (1,321 isolates)
	Number of laboratories participating in Euro-GASP EQA	15	28	Increased by 13 laboratories
	Number of countries participating in the laboratory training	No training in 2012; 13 in 2014	14	Increased by one country
	Proportion of countries reporting epidemiological characteristics (mode of transmission) in Euro-GASP	16/20 (based on transmission data)	17/27 (based on transmission data)	Increased by one country (based on transmission data)
	Completeness of Euro-GASP data for key epidemiological characteristics^a^	85.9% completeness on average; 51.2% for mode of transmission	87.7% completeness on average; 61.6% for mode of transmission	Increased by 1.8% for key variables and by 10.4% for mode of transmission
	Time between Euro-GASP data collection and publication of interim and annual report^b^	12 months	9 months	Report published 3 months sooner
Clinical management	Number of cases of gonorrhoea treatment failure reported in EPIS-STI (using the template)	Only two cases reported in EPIS-STI since publication of 2012 ECDC response plan		True number of treatment failures unknown, but number is an underestimate based on treatment failures reported in literature.
Communication strategy	Number of publications or communications on MDR-NG	Nine peer-reviewed Euro-GASP publications, four in progress. Reports: molecular typing report, annual EQAs and Euro-GASP reports, laboratory capacity survey, training surveys, response plan.		

ECDC: European Centre for Disease Prevention and Control; EPIS-STI: Epidemic Intelligence Information System-Sexually Transmitted Infection; EQA: external quality assessment; Euro-GASP: European Gonococcal Antimicrobial Surveillance Programme; MDR-NG: multidrug-resistant *Neisseria gonorrhoeae*.

^a^ Average percentage completeness taken across all countries for age, sex, mode of transmission (sexual orientation) and site of infection.

^b^ Data collection period is normally during the month of May in the year following isolate collection.

## 2019 ECDC response plan

Decreasing susceptibility to azithromycin, along with increasing reports of ceftriaxone-resistant strains and gonorrhoea treatment failures and of MDR/XDR-NG, made an evaluation, detailed review and update of the 2012 ECDC response plan imperative [7]. The 2019 ECDC response plan was developed by ECDC and the Euro-GASP coordinating hub together with an international expert group to further support EU/EEA countries to implement national strategies and interventions to control the threat of MDR/XDR-NG in a multidisciplinary approach [21]. The 2019 ECDC response plan [21] uses the Tapsall et al. [22] definition of MDR/XDR except that azithromycin has been moved to the Category I list of antibiotics currently used to treat gonorrhoea, and spectinomycin was moved to Category II, which lists antibiotics used less frequently for gonorrhoea treatment [22].

There are three main components in the 2019 ECDC response plan [21]. Firstly, AMR surveillance should be strengthened by expanding participation in Euro-GASP, by improving the completeness of reporting of epidemiological characteristics, timeliness of reporting and representativeness, and by providing training. At the national level, EU/EEA countries are encouraged to have their own national GASP or to ensure participation in Euro-GASP and to collect data on current gonorrhoea treatment. Representativeness in Euro-GASP should be regularly monitored to ensure that the isolates submitted continue to be representative of the AMR and gonorrhoea epidemiological trends across the EU/EEA countries [6]. At a national level, gonococcal AMR data should ideally be from patients representing different patient groups and geographical regions to reflect the distribution of gonorrhoea cases in that country. The ideal proportion of isolates to be cultured is yet to be defined, however it is an important issue to address as the sample size in all settings should be enough to provide a robust estimate of AMR prevalence, particularly in countries with high and increasing numbers of gonorrhoea cases. However, to truly investigate representativeness, the completeness of the epidemiological data reported needs to be sufficiently high. Completeness of data reporting can be improved by ensuring that appropriate data collection, linkage and reporting systems are being used nationally, and by addressing any legal or other regulatory issues that restrict linkage of *N. gonorrhoeae* isolate data to the epidemiological and clinical data of the gonorrhoea cases.

Secondly, a national agreement on adopting the treatment failure case definitions proposed in the 2019 ECDC response plan [21] and an online reporting template for treatment failures should improve the clinical management and monitoring of gonorrhoea treatment failure. Data regarding verified gonorrhoea treatment failures or XDR or ceftriaxone-resistant *N. gonorrhoeae* isolates should be collected and ideally reported promptly to ECDC. Where possible, *N. gonorrhoeae* XDR or ceftriaxone-resistant isolates and those from suspected treatment failures should be shared with the Euro-GASP hub for further verification, including whole genome sequencing, in order to inform the European guideline on the diagnosis and treatment of gonorrhoea [18] and to mitigate subsequent spread. Whole genome sequencing is not only able to confirm an indistinguishable *N. gonorrhoeae* strain before and after treatment and to identify the presence of AMR determinants in treatment failure cases, but additionally has a role in improving AMR surveillance by providing a genomic background to monitor the molecular epidemiology of circulating strains (temporal and geographical changes over time), to detect new and emerging AMR determinants, to ultimately predict AMR and to investigate the genetic relatedness of isolates from national and international outbreaks [23].

Thirdly, the effectiveness of the 2019 ECDC response plan should be monitored at the national and European level (Table 3). More defined and measurable EU/EEA-level indicators will be monitored through routinely collected data and assessed by ECDC and the Euro-GASP hub annually, while data for other indicators will be collected biennially. This was successfully achieved during 2020, when 26 countries provided 2019 indicator data, which enabled progress review over the preceding 2 years and described the impact of COVID-19 during 2020 such as the cancellation of laboratory training and delays in data reporting [24]. 

**Table 3:** Indicators for monitoring drug-resistant *Neisseria gonorrhoeae* at national and EU/EEA levels

Component	Indicator
Strengthen antimicrobial surveillance – EU/EEA level	1.1 Number and proportion of EU/EEA countries participating in Euro-GASP
	1.2 Number of isolates reported through Euro-GASP
	1.3 Number of laboratories participating in Euro-GASP EQA
	1.4 Number of countries and professionals from these countries participating in the ECDC laboratory training
	1.5 Proportion of countries reporting epidemiological characteristics in Euro-GASP
	1.6 Completeness of Euro-GASP data for key epidemiological characteristics
	1.7 Euro-GASP reporting protocol reviewed annually
Strengthen antimicrobial surveillance – national level	1.8 Presence of a national representative isolate collection
	1.9 Number of countries offering national training modules (laboratory and/or clinical)
	1.10 Proportion of all STI clinics (sentinel sites) that have access to culture and antimicrobial susceptibility testing
	1.11 Proportion of all (reported) gonorrhoea cases tested with culture and with antimicrobial susceptibility results available
	1.12 Proportion of patients who received recommended gonorrhoea treatment
Clinical management and treatment failure monitoring	2.1 ECDC contributes to public health aspects of revision of the gonorrhoea patient management guidelines
	2.2 Online reporting template for probable and confirmed gonorrhoea treatment failures developed
	2.3 Number of verified gonorrhoea treatment failures reported to ECDC
Control strategy and communications	3.1 Adoption of national plan to control MDR/XDR gonorrhoea or inclusion in gonorrhoea, STI, sexual health or other relevant strategy
	3.2 Number of visits to ECDC Response Plan website
	3.3 Number of peer-reviewed publications or other communications on antimicrobial resistant *Neisseria gonorrhoeae* from Euro-GASP

ECDC: European Centre for Disease Prevention and Control; EQA: external quality assessment; EU/EEA: European Union/European Economic Area; Euro-GASP: European Gonococcal Antimicrobial Surveillance Programme; MDR: multidrug-resistant; STI: sexually transmitted infection; XDR: extensively drug-resistant.

To monitor the response at the national level, national-level indicators can be used or the EU/EEA level indicators can be adapted to local and national needs.

## Conclusions

Preventing further emergence and spread of AMR in *N. gonorrhoeae* is imperative. The 2019 ECDC response plan should assist in controlling and managing the threat of MDR/XDR-NG in Europe through strengthening AMR surveillance and clinical management including treatment failure monitoring. The effectiveness of the 2019 ECDC response plan should be monitored regularly to identify and address areas for improvement promptly. Control of AMR in *N. gonorrhoeae* is ineffective without a comprehensive approach, therefore the 2019 ECDC response plan needs strong support from comprehensive management and control strategies nationally and internationally, including appropriate STI prevention (e.g. promotion of condom use), diagnostic and testing algorithms (e.g. triple-site testing in men who have sex with men), treatment, test of cure, notification and treatment of partners and robust epidemiological surveillance to identify key groups at risk of gonorrhoea and gonococcal AMR.
